# Supplementary figures and images for: EZH2 Influences mdDA Neuronal Differentiation, Maintenance and Survival
Source: Front Mol Neurosci. 2019 Jan 17;11:491. doi: 10.3389/fnmol.2018.00491 (PMC6344421; doi:10.3389/fnmol.2018.00491)

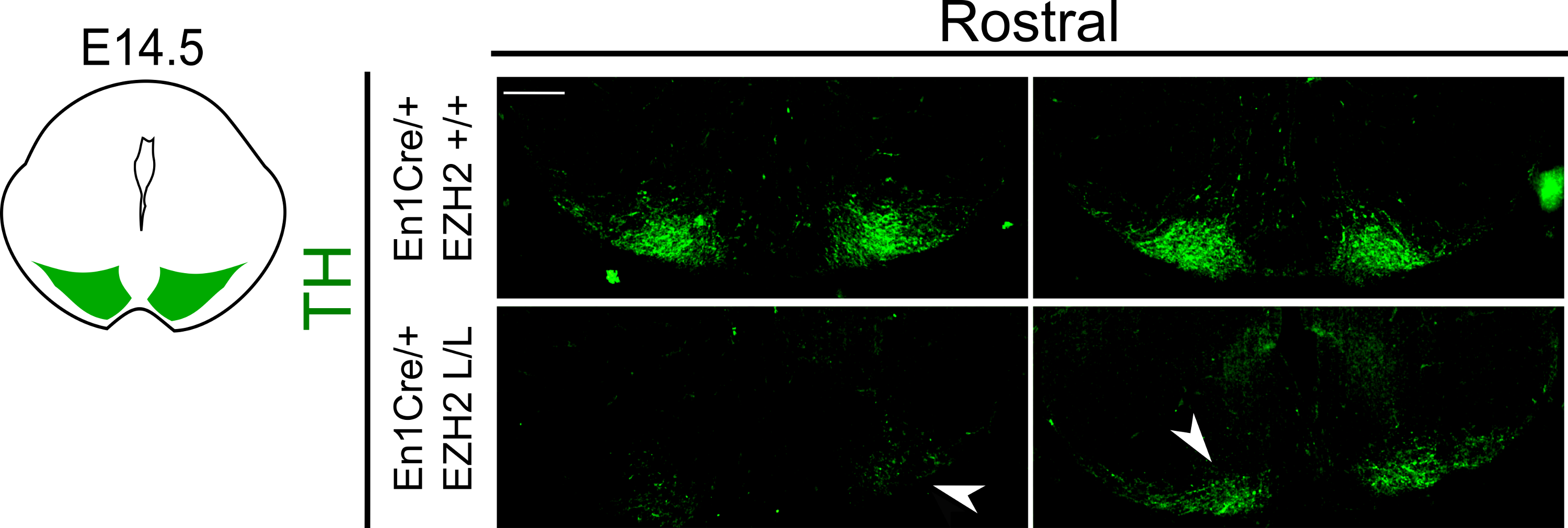

Supplement: Figure S1 — TH expression is reduced in rostral sections of the embryonic midbrain of En1/Ezh2 mutants. Analysis of TH expression in coronal E14.5 midbrain sections by means of immunohistochemistry. Expression of TH is reduced in the rostral sections (white arrowheads). Scale bars = 200 μM. [file Image_1.TIFF]

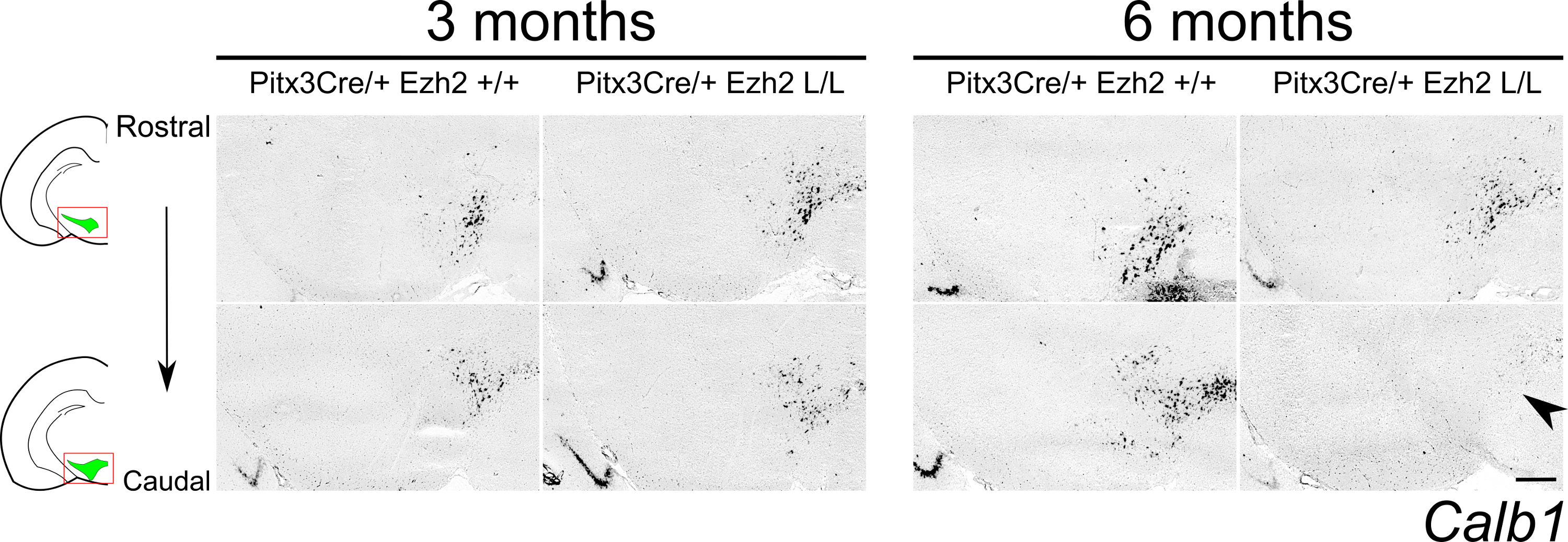

Supplement: Figure S2 — Pitx3Cre driven deletion of Ezh2 induces a progressive loss of Calb1 staining in the VTA. Analysis of the expression of Calb1 in coronal midbrain sections of 3 and 6 months old Pitx3Cre/+; Ezh2+/+ and Pitx3Cre/+; Ezh2 L/L animals by means of in situ hybridization. Expression of Calb1 is lost in the caudal VTA of 6 months old Ezh2 cKO animals (black arrowhead), while expression can still be detected in this location at 3 months (left panel). Scale bars = 300 μM. [file Image_2.TIFF]
